# Mesenteric volvulus associated with exposed barbed suture following laparoscopic TAPP repair: a case report and literature review

**DOI:** 10.1097/RC9.0000000000001098

**Published:** 2026-10-14

**Authors:** Ouyue Wang, Wenchao Zhang, Bo Yang, Jing He

**Affiliations:** Department of General Surgery, Chongqing Fuling Hospital, School of Medicine, Chongqing University, Chongqing, China

**Keywords:** barbed suture, case report, mesenteric volvulus, small bowel obstruction, TAPP repair

## Abstract

**Introduction and importance:**

Barbed sutures are widely used in minimally invasive surgery to facilitate knotless continuous closure. In laparoscopic transabdominal preperitoneal (TAPP) inguinal hernia repair, they are commonly employed for peritoneal flap closure due to their technical efficiency. Although generally considered safe, increasing evidence has linked exposed barbed suture segments to postoperative small bowel complications. Mesenteric volvulus associated with barbed suture entanglement is exceptionally rare and remains poorly characterized.

**Case presentation:**

An 80-year-old man presented with acute abdominal pain and vomiting 26 days after elective laparoscopic TAPP inguinal hernia repair. Computed tomography demonstrated dilated small bowel loops with a characteristic whirl sign, suggesting mesenteric torsion. Emergency laparoscopy revealed a 720° clockwise volvulus of the distal ileum. The volvulus was centered on a focal anchoring point where a residual barbed suture tail had engaged the small bowel serosa. Division of the suture and laparoscopic detorsion may have contributed to the immediate restoration of bowel perfusion without the need for resection. The patient had an uneventful postoperative recovery.

**Clinical discussion:**

Barbed suture–related small-bowel complications are increasingly recognized, most commonly as obstruction associated with adhesions or entanglement. This case suggests that exposed barbed suture segments may also contribute to progressive mesenteric torsion and volvulus formation.

**Conclusion:**

Mesenteric volvulus associated with an exposed barbed suture is a rare but potentially life-threatening complication following a TAPP repair. Meticulous peritoneal closure and careful management of residual suture tails may help reduce the risk of postoperative bowel complications.

## Introduction

Barbed sutures represent a significant technical evolution in minimally invasive surgery^[^1–3^]^. By incorporating unidirectional barbs along the suture filament, these devices eliminate the need for intracorporeal knot tying and distribute tension evenly along the suture line, thereby simplifying laparoscopic suturing and improving procedural efficiency. These advantages are particularly evident in technically demanding steps such as peritoneal closure during laparoscopic transabdominal preperitoneal (TAPP) inguinal hernia repair^[^4,5^]^.

HIGHLIGHTS

- Barbed sutures may be associated with small-bowel volvulus after TAPP repair.
- An exposed suture may serve as a fixed pivot point for mesenteric torsion.
- Volvulus may represent a severe form within the continuum of complications.
- Early laparoscopic intervention can prevent bowel ischemia.
- Proper suture management is critical for prevention.

Since their introduction, barbed sutures have been widely adopted across multiple surgical disciplines, including gastrointestinal, gynecological, and robotic procedures^[^2,3^]^. Accumulating evidence from systematic reviews, meta-analyses, and large cohort studies suggests that barbed sutures provide comparable short-term outcomes to conventional sutures in terms of recurrence, postoperative morbidity, and pain^[^6–9^]^.

However, with increasing utilization, sporadic reports of barbed suture–related small bowel complications have emerged, including small bowel obstruction, internal herniation, and closed-loop obstruction^[^6,10–20^]^. Although the overall incidence appears low, the consistency of intraoperative findings across different surgical specialties suggests a reproducible mechanical phenomenon rather than isolated coincidence.

Most reported cases involve focal anchoring of exposed barbed suture segments to adjacent bowel or mesentery, resulting in obstruction^[^6,13^]^. True mesenteric volvulus, characterized by axial rotation of the bowel around its mesenteric axis, is far less frequently described but represents a potentially severe manifestation within this spectrum of complications^[^14,15^]^.

Herein, we report a case of delayed 720° mesenteric volvulus occurring 26 days after TAPP repair using a barbed suture for peritoneal closure. In addition to describing the clinical course, we integrate existing evidence and propose a biomechanical model to explain the progression from initial serosal engagement to delayed axial torsion. This case has been reported in line with the SCARE Guideline Checklist 2025^[^21^]^.

## Case presentation

An 80-year-old man with no significant past medical history presented with symptomatic bilateral inguinal hernias. Preoperative imaging and intraoperative findings demonstrated bilateral indirect inguinal hernias, with the right side more prominent than the left. The hernia sacs contained reducible small bowel and preperitoneal fat without evidence of incarceration or strangulation. The approximate defect sizes were 2.5 cm on the right and 1.5 cm on the left. Preoperative evaluation was unremarkable, and elective laparoscopic TAPP repair was performed in accordance with established international guidelines^[^22^]^.

The patient was placed in the supine position under general anesthesia. A standard three-port laparoscopic TAPP approach was used, consisting of a 10-mm infraumbilical camera port and two 5-mm working ports placed in the bilateral lower quadrants along the midclavicular lines. Pneumoperitoneum was established using carbon dioxide insufflation and maintained at 12 mm Hg throughout the procedure. The peritoneal incision was created approximately 2 cm above the internal inguinal ring to allow adequate exposure of the myopectineal orifice and sufficient peritoneal flap mobilization for tension-free closure. A lightweight polypropylene mesh was selected because it would be placed entirely within the preperitoneal space and separated from the abdominal viscera by peritoneal closure, in accordance with standard TAPP practice. No bowel exposure to the mesh was present during the initial repair; therefore, anti-adhesive composite mesh was not considered necessary. Mesh fixation was performed selectively with absorbable, atraumatic fixation at the medial Cooper’s ligament region, while extensive lateral fixation was intentionally avoided to minimize the risk of nerve injury within the triangle of pain and chronic postoperative pain. Peritoneal closure was performed using a continuous running 3-0 absorbable barbed suture (V-Loc™, Medtronic). The terminal tail was trimmed laparoscopically to a residual length of approximately 5 mm. No additional fixation clips or knot reinforcement were used. The closure technique followed the manufacturer’s standard knotless running closure principle commonly adopted in TAPP repair.

The immediate postoperative course was uneventful. The patient was discharged on postoperative day 3 and resumed normal activities. The 3-day postoperative hospitalization was primarily due to advanced age, routine institutional observation protocols, and monitoring to ensure adequate pain control and the return of bowel function. During this period, the patient remained hemodynamically stable, without fever, tachycardia, or signs of early postoperative complications.

On postoperative day 26, the patient presented with acute diffuse abdominal pain, nausea, and repeated vomiting. Physical examination revealed abdominal distension and tenderness without peritoneal signs. Laboratory tests showed mild leukocytosis without metabolic acidosis.

Initial abdominal radiography demonstrated multiple dilated small bowel loops with air-fluid levels, suggesting mechanical small bowel obstruction. Given persistent abdominal pain, vomiting, and concern for possible strangulation or mesenteric ischemia, contrast-enhanced computed tomography was subsequently performed for further evaluation. Contrast-enhanced computed tomography demonstrated dilated small bowel loops and a characteristic whirl sign, indicating mesenteric torsion. Three-dimensional CT angiography was also used to better evaluate mesenteric vascular rotation and bowel perfusion status. Three-dimensional CT angiography further confirmed a rotational configuration of the mesenteric vessels, with no evidence of pneumatosis intestinalis or portal venous gas (Fig. 1).

**Figure 1.**
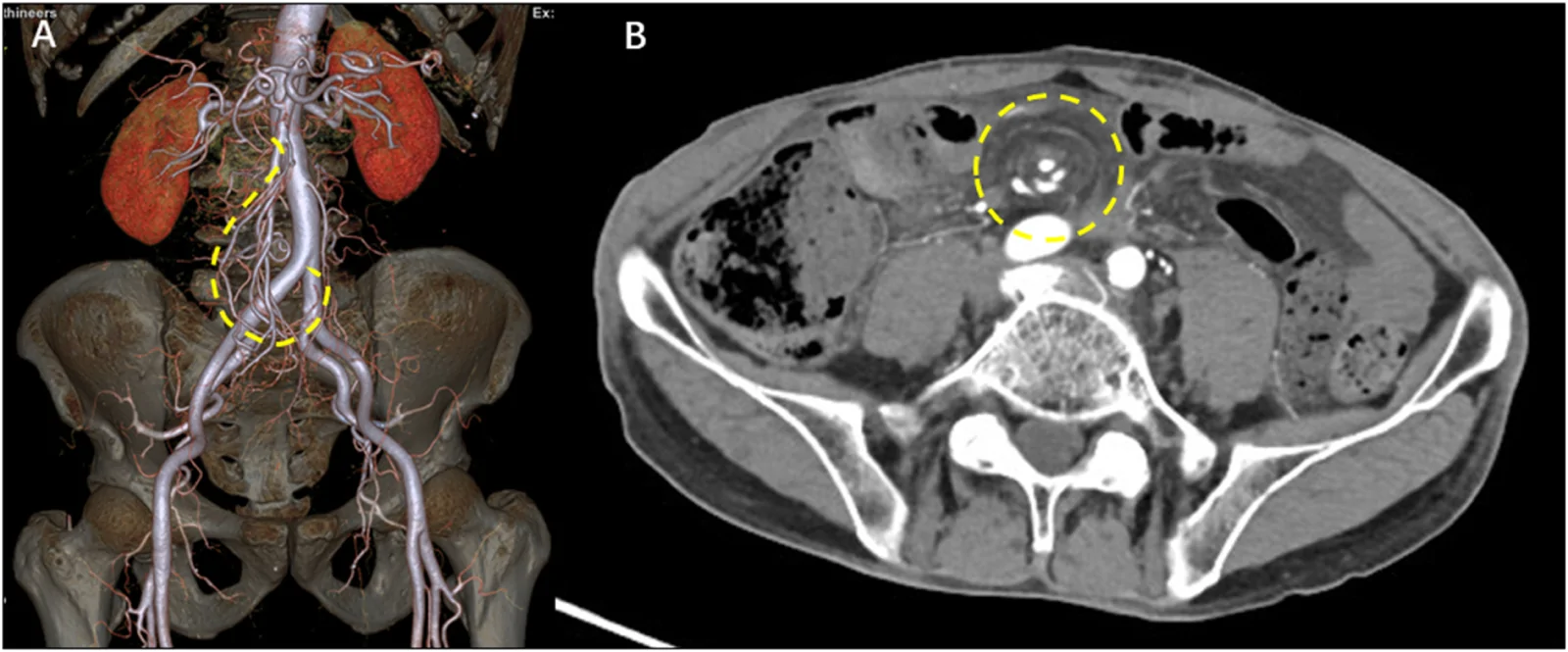
Preoperative contrast-enhanced computed tomography (CT) demonstrating characteristic imaging findings of small bowel volvulus. (A) Three-dimensional reconstruction of mesenteric arteries from CT angiography, demonstrating rotational configuration of the mesenteric vessels, further confirming the presence of mesenteric torsion. (B) Axial arterial-phase CT image showing the classic “whirl sign” of the mesentery, characterized by spiral twisting of the mesenteric fat and vessels, suggestive of small bowel volvulus.

These imaging findings supported the diagnosis of small bowel volvulus prior to surgical intervention.

Because the patient remained hemodynamically stable without diffuse peritonitis or radiological evidence of bowel perforation, a laparoscopic exploratory approach was considered feasible and appropriate. Given these findings, emergency laparoscopy was performed. Approximately 60 cm proximal to the ileocecal valve, the distal ileum was found to have undergone a 720° clockwise volvulus around its mesenteric axis. The mesentery appeared congested but viable, with no evidence of generalized adhesions. A focal anchoring point was identified between the ileal serosa and an exposed tail of barbed suture from the previous peritoneal closure. The barbed segment had engaged the serosa, acting as a fixed pivot point for torsion (Fig. 2). No mesh migration, mesh exposure, or direct mesh-related bowel entrapment was identified intraoperatively.

**Figure 2.**
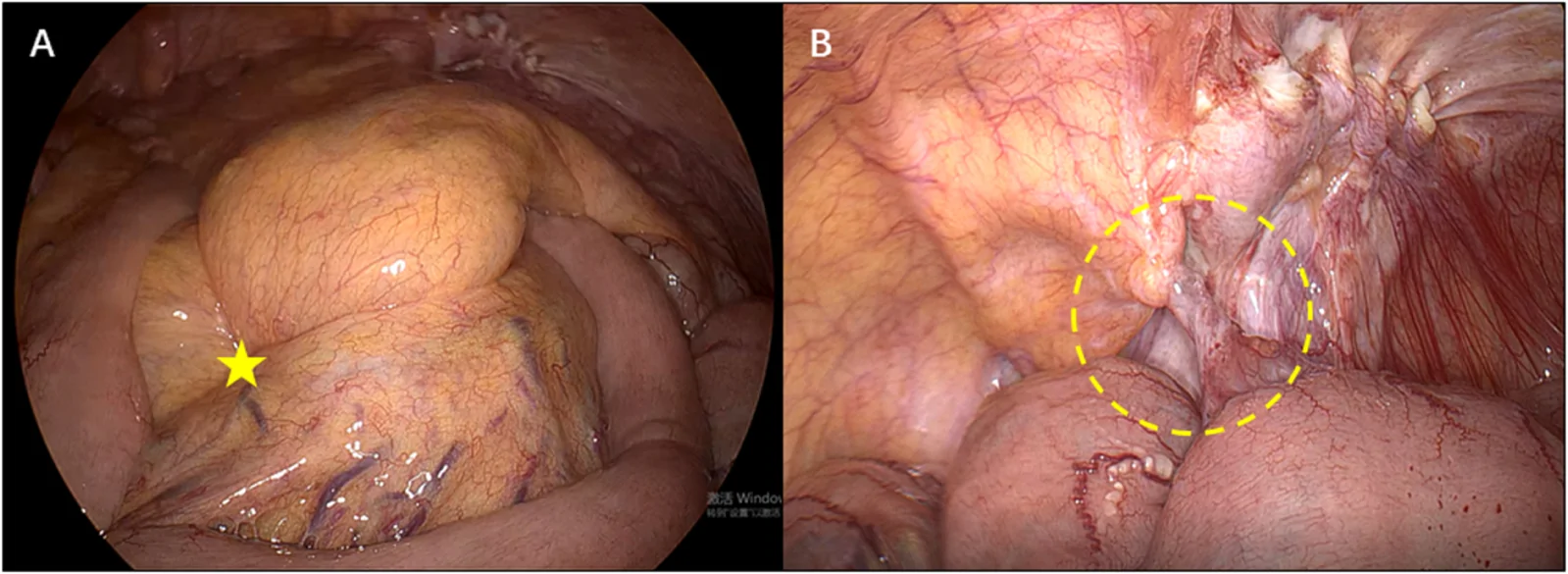
Intraoperative laparoscopic findings demonstrating small-bowel volvulus associated with entrapment of the bowel by a residual barbed suture following transabdominal preperitoneal (TAPP) inguinal hernia repair. (A) Laparoscopic exploration revealed torsion of the distal ileum with congestion and dilatation of the mesentery, consistent with small-bowel volvulus. (B) Further inspection identified a focal anchoring point where a residual barbed suture tail from the peritoneal closure had engaged the small bowel approximately 60 cm proximal to the ileocecal valve, potentially contributing to mesenteric torsion. After division of the barbed suture, the volvulus was successfully reduced and bowel perfusion was restored.

The suture tail was sharply divided and removed, followed by laparoscopic detorsion. Bowel perfusion improved promptly, with restoration of normal color and mesenteric pulsation. No bowel resection was required. Inspection of the peritoneal closure revealed no additional exposed suture segments.

The postoperative course was uneventful, and the patient was discharged on postoperative day 5. At 2-month follow-up, he remained asymptomatic, with no evidence of recurrent obstruction or hernia recurrence.

## Discussion

A focused literature review was performed using the PubMed, Web of Science, and Embase databases. The search terms included “barbed suture,” “small bowel obstruction,” “volvulus,” “TAPP,” and “laparoscopic surgery.” Only English-language case reports and case series describing postoperative bowel complications associated with barbed sutures were included. Relevant references from retrieved articles were also manually screened.

A synthesis of previously reported cases is presented in Table 1, demonstrating consistent clinical and intraoperative patterns across multiple surgical disciplines. Barbed sutures are widely used in minimally invasive surgery due to their ability to facilitate knotless closure and maintain uniform tension. In laparoscopic TAPP repair, they offer technical efficiency and have demonstrated comparable short-term outcomes to conventional sutures^[2,3,6,7,9]^. However, increasing reports have described postoperative small bowel complications associated with exposed barbed suture segments^[^6,10–23^]^.

**Table 1:** Published cases of barbed suture–related small bowel complications in minimally invasive surgery.

Author (year)	Surgery	Interval	Complication type	Mechanism	Bowel resection
Zheng (2021)^[^10^]^	TAPP	14 days	SBO	Suture tail entrapment	No
Wang (2021)^[^11^]^	TAPP	10–20 days	SBO + perforation	Bowel hooking by exposed barb	Yes
Tagliaferri (2018)^[^12^]^	TAPP	15 days	SBO	Adhesion to suture tail	No
Clapp (2020)^[^6^]^	Various	Variable	SBO	Barbed anchoring effect	Mixed
Santucci (2024)^[^13^]^	Sleeve/RYGB	2–3 weeks	SBO	Loop entrapment	No
Takagi (2024)^[^14^]^	Sacrocolpopexy	30 days	SBO	Suture–bowel entanglement	No
Filser (2015)^[^15^]^	TAPP	20 days	Volvulus	Fixed pivot point by suture	Yes
Chen (2017)^[^16^]^	Hysterosacropexy	2 days	SBO	Early entrapment	No
Sakata (2015)^[^17^]^	Rectopexy	NR	SBO	Direct bowel adhesion	No
Sarhan (2023)^[^18^]^	RYGB	Early	SBO	Free suture end entrapment	No
Jang (2017)^[^19^]^	Gastrectomy	7–10 days	SBO	Serosal hooking	No
Lee (2015)^[^20^]^	Myomectomy	1–2 weeks	SBO	Barbed suture adhesion	No
Pajtak (2025)^[^7^]^	Various	Variable	SBO	Consistent anchoring	Mixed
Gee (2023)^[^23^]^	Laparoscopy	Early	SBO	Mechanical obstruction	No

Among the reported cases summarized in Table 1, most complications presented between postoperative day 10 and day 30, with small bowel obstruction being the predominant manifestation. True mesenteric volvulus was reported only in a minority of cases.

Among the reported cases summarized in Table 1, most complications presented between postoperative days 10 and 30, with small-bowel obstruction being the predominant manifestation. True mesenteric volvulus was reported in only a minority of cases.

A focused review of the literature identified more than 30 reported cases of barbed suture–related small bowel complications across multiple surgical specialties, including TAPP repair, bariatric surgery, colorectal surgery, and robotic pelvic procedures. Most cases present with delayed small-bowel obstruction, typically occurring 10–30 days postoperatively. Intraoperative findings are highly consistent, characterized by focal anchoring of an exposed barbed segment to the bowel or mesentery, absence of diffuse adhesions, and rapid resolution following division of the suture^[^6,15,17^]^. Compared with previously reported cases, several aspects make the present case clinically distinctive. First, the patient developed a high-grade 720° mesenteric volvulus rather than a simple adhesive obstruction. Second, symptom onset occurred nearly 4 weeks after surgery, supporting the possibility of a delayed and progressive pathological process rather than immediate postoperative entanglement alone. Third, the intraoperative findings allowed the formulation of a possible biomechanical progression model linking exposed barbed suture engagement, focal fixation, progressive rotational traction, and eventual volvulus formation. Most previously reported barbed suture–related complications have presented as simple obstruction or focal adhesion, whereas true mesenteric volvulus appears considerably less common. The present case provides important insight into this phenomenon. Intraoperatively, a single exposed barbed suture tail engaged the ileal serosa, forming a fixed pivot point around which a 720° mesenteric volvulus developed. No alternative causes, such as adhesive disease or mesh-related entrapment, were identified. These findings support the possibility that exposed barbed suture segments may contribute to a reproducible mechanical interaction rather than representing nonspecific postoperative adhesions alone.

From a mechanistic perspective, these observations raise the possibility that barbed suture–related bowel complications may represent a progressive spectrum rather than entirely distinct pathological entities. Nevertheless, this proposed biomechanical model remains speculative and requires further validation through additional clinical and experimental studies. Initial serosal engagement by an exposed barbed segment may lead to intermittent or partial obstruction. Persistent fixation can evolve into a closed-loop configuration, and under ongoing peristaltic forces, progressive axial rotation may ultimately result in volvulus. This delayed presentation may be explained by the dynamic process of peritoneal healing and adhesion formation. Mesothelial repair and fibrin remodeling occur over several weeks following surgery, during which transient serosal contact may evolve into stable anchoring points^[^24–27^]^. Nevertheless, the consistency of intraoperative findings across previously reported cases, together with the known asymmetric resistance properties of barbed sutures, provides indirect support for this proposed biomechanical mechanism. A schematic representation of this proposed biomechanical hypothesis is shown in Figure 3.

**Figure 3.**
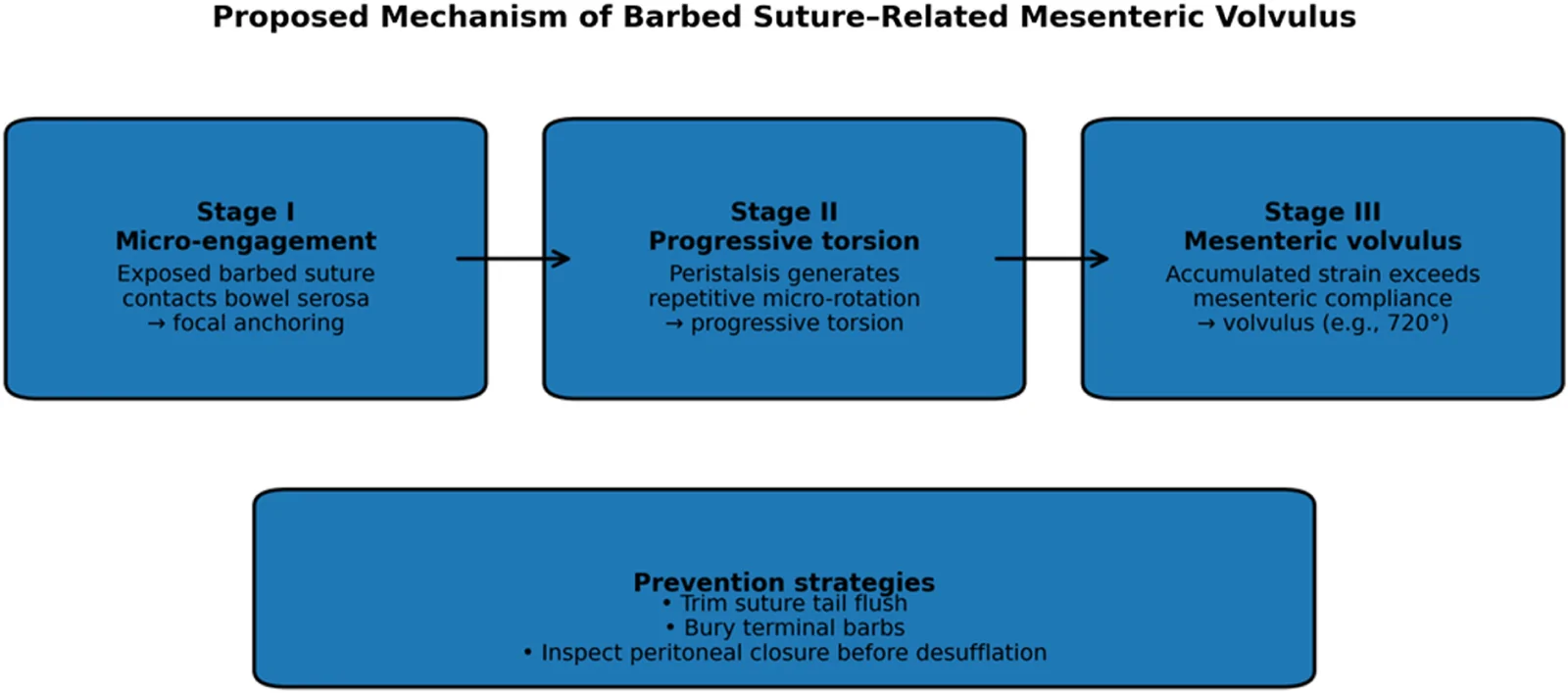
Proposed biomechanical mechanism of barbed suture–related mesenteric volvulus.

**Stage I:** An exposed barbed suture tail may engage the small bowel serosa, creating a focal anchoring point.

**Stage II**: Physiological peristalsis may generate repetitive micro-rotational forces around the anchored segment.

**Stage III**: Accumulated torsional forces may eventually contribute to axial rotation and volvulus formation.

Preventive strategies include trimming the suture tail flush, burying terminal barbs, and careful inspection of peritoneal closure.

From a biomechanical perspective, the unidirectional barbed structure generates asymmetric resistance to movement, which may facilitate progressive tightening once tissue engagement occurs^[^28,29^]^. The unidirectional design of barbed sutures may contribute to progressive tissue fixation. Once tissue engagement occurs, reverse displacement becomes more difficult, potentially allowing the gradual accumulation of torsional force over time.

Clinically, recognition of this potentially serious complication is critical. Volvulus carries a higher risk of bowel ischemia compared with simple obstruction, and early surgical intervention is essential. In the present case, prompt laparoscopic exploration enabled detorsion and preservation of bowel viability, thereby avoiding resection.

At present, available evidence does not support complete avoidance of barbed sutures in TAPP repair, given their well-established technical advantages and generally favorable safety profile. Instead, this case highlights the importance of meticulous technique modifications. Preventive strategies may include minimizing exposed suture tail length, burying terminal segments when feasible, carefully inspecting the peritoneal closure before desufflation, and considering conventional non-barbed absorbable sutures in selected high-risk situations. In addition, conventional non-barbed absorbable sutures remain a well-established and reliable option for peritoneal closure during TAPP repair and may be preferred by some surgeons, particularly in situations where concern exists regarding exposed barbed segments.

This study has limitations. As a single case report, it cannot establish causality or estimate true incidence. In addition, the literature may be subject to reporting bias. Nevertheless, the consistency of findings across reports and a plausible explanation support the clinical relevance of this complication. An additional limitation is the relatively short follow-up duration of 2 months, which may be insufficient to fully evaluate long-term recurrence, adhesion-related complications, or delayed bowel obstruction.

In conclusion, barbed suture–related mesenteric volvulus represents a serious complication following TAPP repair. Surgeons should be aware of this potential risk and adopt careful suture management techniques to minimize postoperative small-bowel complications.

## Conclusion

This case highlights a rare but clinically important postoperative complication associated with an exposed barbed suture following laparoscopic TAPP repair. Although barbed sutures remain effective and widely used, meticulous peritoneal closure and careful management of residual suture tails are essential to minimize the risk of delayed bowel complications. Early recognition and prompt laparoscopic intervention may help prevent bowel ischemia and avoid intestinal resection.

## Data Availability

The data supporting the findings of this case report are available from the corresponding author upon reasonable request, subject to patient confidentiality and applicable ethical restrictions.
